# Supplementary material for: Pollution and health risk assessment of rare earth elements in Citrus sinensis growing soil in mining area of southern China
Source: PeerJ. 2023 Jun 6;11:e15470. doi: 10.7717/peerj.15470 (PMC10252884; doi:10.7717/peerj.15470)

Supplementary materials

Table S1 The relationship among soil factors by Pearson correlation analysis

|  | Fe_2_O_3_ | Na_2_O | K_2_O | pH | TOC |
| --- | --- | --- | --- | --- | --- |
| Fe_2_O_3_ | 1 |  |  |  |  |
| Na_2_O | -0.477** | 1 |  |  |  |
| K_2_O | -0.642** | 0.870** | 1 |  |  |
| pH | -0.36 | 0.263 | 0.319 | 1 |  |
| TOC | 0.17 | -0.349 | -0.449* | 0.155 | 1 |

Fig. S1 The schematic diagram of the experimental design


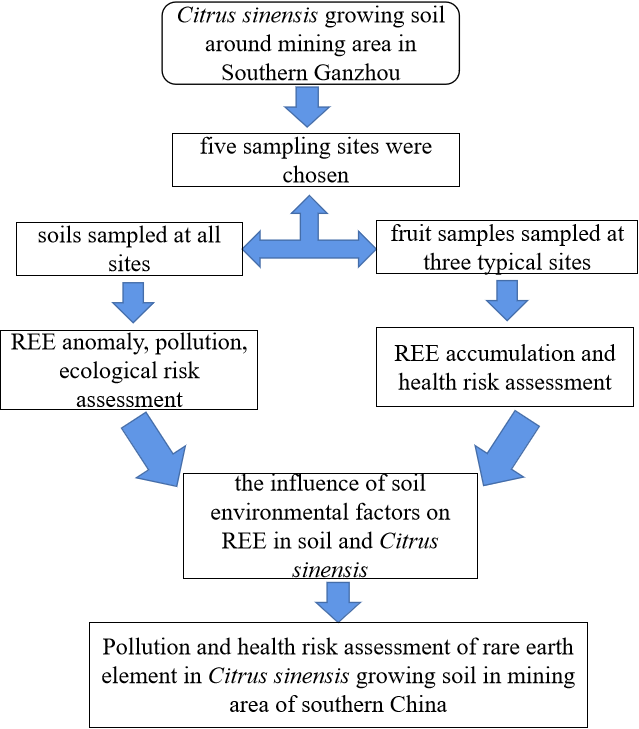

Supplement: Supplemental Information 1 [file peerj-11-15470-s001.docx]
